# EST mining identifies proteins putatively secreted by the anthracnose pathogen *Colletotrichum truncatum*

**DOI:** 10.1186/1471-2164-12-327

**Published:** 2011-06-23

**Authors:** Vijai Bhadauria, Sabine Banniza, Albert Vandenberg, Gopalan Selvaraj, Yangdou Wei

**Affiliations:** 1 Department of Biology, University of Saskatchewan, 112 Science Place, Saskatoon, SK S7N 5E2 Canada; 2 Crop Development Centre, University of Saskatchewan, 51 Campus Drive, Saskatoon, SK S7N 5A8 Canada; 3 Plant Biotechnology Institute, National Research Council of Canada, 110 Gymnasium Place, Saskatoon, SK S7N 0W9 Canada

## Abstract

**Background:**

*Colletotrichum truncatum *is a haploid, hemibiotrophic, ascomycete fungal pathogen that causes anthracnose disease on many economically important leguminous crops. This pathogen exploits sequential biotrophic- and necrotrophic- infection strategies to colonize the host. Transition from biotrophy to a destructive necrotrophic phase called the biotrophy-necrotrophy switch is critical in symptom development. *C. truncatum *likely secretes an arsenal of proteins that are implicated in maintaining a compatible interaction with its host. Some of them might be transition specific.

**Results:**

A directional cDNA library was constructed from mRNA isolated from infected *Lens culinaris *leaflet tissues displaying the biotrophy-necrotrophy switch of *C. truncatum *and 5000 expressed sequence tags (ESTs) with an average read of > 600 bp from the 5-prime end were generated. Nearly 39% of the ESTs were predicted to encode proteins of fungal origin and among these, 162 ESTs were predicted to contain N-terminal signal peptides (SPs) in their deduced open reading frames (ORFs). The 162 sequences could be assembled into 122 tentative unigenes comprising 32 contigs and 90 singletons. Sequence analyses of unigenes revealed four potential groups: hydrolases, cell envelope associated proteins (CEAPs), candidate effectors and other proteins. Eleven candidate effector genes were identified based on features common to characterized fungal effectors, i.e. they encode small, soluble (lack of transmembrane domain), cysteine-rich proteins with a putative SP. For a selected subset of *CEAPs *and candidate effectors, semiquantitative RT-PCR showed that these transcripts were either expressed constitutively in both *in vitro *and *in planta *or induced during plant infection. Using potato virus X (PVX) based transient expression assays, we showed that one of the candidate effectors, i. e. contig 8 that encodes a cerato-platanin (CP) domain containing protein, unlike CP proteins from other fungal pathogens was unable to elicit a hypersensitive response (HR).

**Conclusions:**

The current study catalogues proteins putatively secreted at the *in planta *biotrophy-necrotrophy transition of *C. truncatum*. Some of these proteins may have a role in establishing compatible interaction with the host plant.

## Background

**Figure 1:**
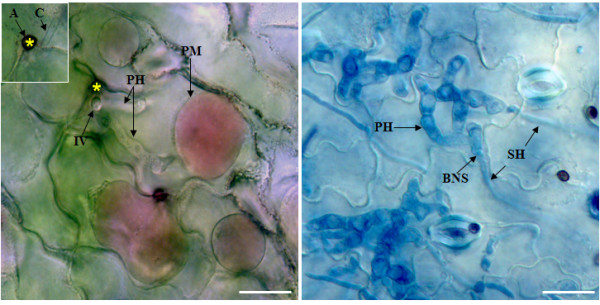
**Histochemical analysis of *C. truncatum *infected lentil leaflet tissues.** (A) Plasmolyzed (0.8 M NaNO_3_) infected cells of lentil cultivar 'Eston' stained with Neutral Red (0.1% in water) vital dye show that the intracellular primary hyphae of *C. truncatum *do not form any plant-pathogen interface and remain separated during the biotrophic phase. C, conidia; A (*), appressorium; IV, infection vesicle; PH, primary hyphae; and PM, plasma membrane. (B) Infected leaflet cells of Eston display the transition from the biotrophic phase to necrotrophic phase of *C. truncatum*. BNS, biotrophy-necrotrophy switch; and SH, secondary hyphae. Bars = 10 μm.

*Colletotrichum truncatum *(Schwein.) Andrus W.D. Moore causes anthracnose disease of many leguminous species, including lentil (*L. culinaris *Medik.), soybean (*Glycine max *(L) Merr.), fababean (*Vicia faba *L.), and pea (*Pisum sativum *L.) [1]. This fungal pathogen employs a bi-phasic hemibiotrophic infection strategy to colonize lentil plants. The anthracnose infection is initiated by the attachment of conidia to aerial parts of the host plant. Conidia germinate immediately after adhesion and differentiate to form infection structures called appressoria devoted to mechanical penetration. After the appressorium has been formed, a thin infection or penetration peg emerging from the base of the appressorium pierces the host cuticle and cell wall, and grows in between the plant cell wall and plasma membrane. It differentiates into large, bulbous invasive primary hyphae that are biotrophic in nature. These hyphae are likely to interact with the host plasma membrane but pull away after plasmolysis (Figure 1A), indicating either initial or weak host-pathogen association. This situation differs from that of haustoria (analogous to primary hyphae) and the biotrophic invasive hyphae produced by obligate biotrophic fungi, such as powdery mildews and rusts, and other hemibiotrophs like *Magnaporthe oryzae*, the causal agent of rice blast, where the host plasma membrane remains adhered to fungal structures upon plasmolysis [2]. The primary hyphae of *C. truncatum *are entirely confined to the first infected epidermal cell throughout the biotrophic phase [3]. Thereafter, the fungus switches to the necrotrophic phase that is associated with the production of thin secondary hyphae that ramify intra- and inter-cellularly, killing and macerating host tissues by hydrolytic enzymes ahead of infection. Therefore, the biotrophy-necrotrophy switch (Figure 1B) is critical in anthracnose development. During the transition, *C. truncatum *probably secretes a range of proteins to establish a compatible interaction with its host, including some that may exclusively be involved in switching the pathogen to the necrotrophic phase.

*In silico *analyses of whole genome sequences have predicted that fungal and oomycete phytopathogens possess large repertoires of secretory proteins, which constitute 7 to 10% of predicted proteomes of *M. oryzae *[4], *Phytophthora sojae, P. ramorum *[5] and *Ustilago maydis *[6]. These secretory proteins play diverse roles in virulence and pathogenicity, and interactions with host cells. Secreted hydrolytic enzymes like cell wall degrading enzymes contribute to penetration of the plant cuticle and cell wall, and to tissue maceration during the onset of the necrotrophic stage [5]. Other secretory proteins, such as chitin deacetylase and proteins that bind pathogen cell wall components like chitin binding protein Avr4 of *Cladosporium fulvum*, modify the pathogen cell wall and are critical in evading the host defense surveillance system [7,8]. Secreted effector proteins alter biochemical, physiological, and morphological processes in host plant cells, thereby facilitating infection (virulence factors) or triggering defense responses (avirulence factors or elicitors) [9]. Some of these proteins are active in the apoplast where they may interfere with host plant defense processes, e.g. by inhibiting plant proteases and lytic enzymes. Other fungal effector proteins enter into host cells, though the mechanism of entry of effectors is still a mystery. However, the role of RXLR (arginine, any amino acid, leucine, arginine) and dEER motifs (aspartate [less conserved], glutamate, arginine) in host cell targeting of oomycete effectors has been established [10]. Recently, Kale and colleagues [11] have shown that fungal effectors contain functional variants of RXLR motifs, and that the oomycete and fungal RXLR and dEER motifs bind to host cell surface phosphatidylinositol-3-phosphate. This binding may facilitate the uptaking of effectors through endocytosis.

One of the functions of fungal and oomycete effectors (cytoplasmic effectors) is to disrupt the host signal transduction pathways that mediate defense responses. Until now, only one effector, *CgDN3 *has been identified from *Colletotrichum*. This effector averts HR cell death during the biotrophic phase of infection and is essential for *C. gloeosporioides *pathogenicity on *Stylosanthes guianensis *[12]. Studies conducted to date have investigated the secretome at different stages of the infection process, including penetration (appressorial morphogenesis), biotrophic and necrotrophic stages. Kleeman et al. [13] identified 26 putative soluble secretory proteins from a cDNA library prepared from mature appressoria of *C. higginsianum *grown *in vitro*. Sixteen candidate effector proteins were discovered from *Venturia inaequalis*, the causal agent of apple scab, on the basis of their small size (< 400 amino acids in length when mature), presence of cysteine residues and of a putative SP [14]. Using a yeast signal sequence trap screen, Krijger et al. [15] identified secreted proteins from *C. graminicola *mycelia grown *in vitro *on corn cell walls and leaf extract. Recently, Takahara and colleagues [16] developed a fluorescence-activated cell sorting method to purify the intracellular biotrophic hyphae from *C. higginsianum *from homogenates of infected *Arabidopsis *leaves and constructed a biotrophy-specific cDNA library. Mosquera et al. [17] identified four biotrophy associated secreted proteins from the invasive hyphae of *M. oryzae *by interaction transcriptome analysis. So far, few reports have addressed the critical switch point of the hemibiotrophic infection process. Only one gene encoding a switch regulator, CLTA1 (*C. lindemuthianum *transcriptional activator 1) from *C. lindemuthianum*, has been identified. Mutants harboring disrupted *CLTA1 *were unable to switch to the necrotrophic phase. This transcriptional factor is indispensable for pathogenicity on common bean [18].

The objective of the current study was to identify proteins putatively secreted during the *in planta *biotrophy-necrotrophy switch of *C. truncatum*. To catalogue such proteins, we have generated 5000 ESTs from a directional cDNA library constructed from susceptible Canadian lentil cultivar 'Eston' inoculated with *C. truncatum *isolate CT-21. By biocomputational analyses of these ESTs, we identified eleven *C. truncatum *candidate effector genes. *In planta *functional analysis of three effectors was carried out using PVX-based agroinfiltration in tobacco leaves.

## Results

### Generation of *C. truncatum* ESTs

**Figure 2:**
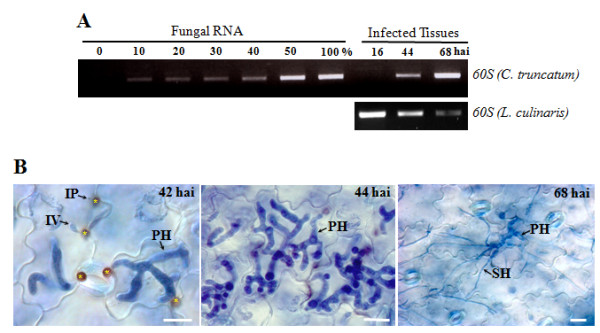
**Evaluation of plant and fungal contents in infected lentil leaflet tissues, and cytological analysis of an infection time-course. **(A) Semiquantitative RT-PCR amplification of the *C. truncatum *and *L. culinaris 60S *ribosomal transcripts in the appressorial penetration phase (16 hai), biotrophic phase (44 hai) and necrotrophic phase (68 hai). Fungal *60S *transcripts were also qualitatively assessed in control mixtures, which were obtained by combining 0, 10, 20, 30, 40, 50, and 100% RNA from fungal mycelium into RNA from mock-inoculated leaflets. Twenty six cycles were used for amplification. (B) *In planta *infection time- course. *C. truncatum *infected lentil leaflets cultivar 'Eston' at 42-44 hai represent the biotrophic phase characterized by large intracellular primary hyphae and the necrotrophic phase at 68 hai. *Appressorium, IP, Infection peg. Bars = 10 μm.

Excised leaflets of Canadian lentil cultivar 'Eston' inoculated with isolate CT-21 of *C. truncatum *were collected and examined microscopically to ensure that the pathogen was at the biotrophic to necrotrophic transition stage where secondary hyphae had become visible (Additional file 1). This corresponded to 48-56 hours after inoculation (hai) and we referred to this period of infection as the biotrophy-necrotrophy switch. Total RNA was extracted, and poly A^+ ^mRNA was isolated and used for construction of a directional cDNA library. Using 5'-end single pass sequencing, 5000 ESTs were generated with an average read length of more than 600 nucleotides. BLASTX analysis with a cutoff *E *value ≤ 10^-5 ^identified 57% as plant sequences, 39% as fungal sequences and the remainder (4%) as unassignable. The high abundance of fungal transcripts was further verified by semiquantitative RT-PCR using the fungal *60S *ribosomal gene in an infection time-course study that included control mixtures (RNA mixtures were obtained by combining 0, 10, 20, 30, 40, 50 and 100% RNA from fungal mycelia into RNA from mock-inoculated lentil leaflets). An accumulation of around 40% of fungal *60S *ribosomal transcripts, which, in turn, reflect the fungal RNA content, was predicted during the biotrophy-necrotrophy switch. The abundance of plant origin transcripts had declined with fungal proliferation in infected host tissues as shown by RT-PCR analysis of *L. culinaris 60S *ribosomal gene in the same infection time-course used for assessing the fungal biomass (Figure 2A).

### The fungal transcriptome contains sequences encoding putative secretory proteins

Fungal sequences (1934 ESTs) were analyzed for features indicative of secreted proteins. Because of directional cloning, we could analyze the coding sequences from the 5'-end. ORF finder, and SignalP and iPSORT algorithms were exploited to deduce ORFs and SPs within the ORFs. One hundred sixty-two predicted ORFs (8.37% of the total fungal ESTs) were predicted to encode proteins with N-terminal SPs. All 162 ESTs were deposited in the NCBI GeneBank EST database (accession numbers HO663580 to HO663741). Using ContigExpress software (Invitrogen), these ORFs could be assembled into 32 contigs and 90 singletons, resulting in a total of 122 unigenes. Clone IDs belonging to various contigs are listed in the additional file 2. The average G+C content of these unigenes was around 59%. We refer to the deduced proteins encoded by these unigenes as putative secretory proteins.

We also employed BLASTX and ORF finder algorithms to investigate whether the first methionine within the amino acid translation represented the true N-terminal methionine to confirm the ORF of selected unigenes. The ORFs were then queried against the NCBI non-redundant protein database using BLASTP algorithm. Fungal effectors are most likely to be small, soluble, extracellular secreted proteins that do not become cross-linked into the fungal cell wall [13]. Therefore, predicted ORFs from these unigenes were screened for the size of the encoded polypeptide chain and the presence of transmembrane domains, cysteine residues, transmembrane domain and glycosylation sites, including glycosyl-phosphatidylinositol (GPI) modification. However, with the steady increase in the number of fungal phytopathogen genomes being sequenced, the likelihood that orthologs found in other species is increasing. Hence, some candidate effectors were identified based on orthologs in other phytopathogens. Comparison of the protein sequences encoded by the unigenes to the currently annotated databases and their sequence analyses revealed four groups. The most highly represented group comprised hydrolytic enzymes, which included 63 unigenes (52%), followed by 36 CEAPs (30%), 11 candidate effector proteins (9%) and 11 proteins (9%) classified as "other proteins". Based on these analyses, a total of 43 unigenes were predicted to encode either proteins with transmembrane domain(s) or GPI addition signal. Among them, six were grouped with hydrolases. The remaining 79 unigenes encoded putatively soluble secretory proteins, including hydrolases. A list of the clone IDs, the top hit for each sequence and the corresponding BLAST score are compiled in Table 1. An *E *value cutoff ≤ 10^-5 ^was used to annotate these unigenes. Four sequences had no match at ≤ 10^-5 ^but contained conserved signatures. Therefore, they were classified according to sequence characteristics belonging to corresponding groups and listed in Table 1.

**Table 1:** *C. truncatum *unigenes encoding secretory proteins

Unique sequence ID	Accession	Putative function	Organism	*E *value
***Cell envelop associated protein***				
Contig 1	XP_002144203	GPI anchored serine-threonine rich protein	*Penicillium marneffei*	9e-11
Contig 2	XP_001269791	GPI anchored serine-threonine rich protein	*Aspergillus clavatus*	1e-06
Contig 3	XP_002148880	GPI anchored protein, putative	*Penicillium marneffei*	1e-14
Contig 4	XP_002850839	GPI anchored serine-rich protein	*Microsporum canis*	5e-11
Ct21-4350	XP_002144203	GPI anchored serine-threonine rich protein	*Aspergillus fumigatus*	8e-11
Ct21-949	EEY14502	GPI-anchored cell wall organization protein Ecm33	*Verticillium albo-atrum*	4e-49
Ct21-1020	EEY23888	GPI transamidase component Gpi16	*Verticillium albo-atrum*	4e-100
Ct21-3268	XP_750946	CFEM domain-containing protein	*Glomerella graminicola*	4e-40
Contig 7	CAQ16271	Hypothetical protein	*Glomerella graminicola*	1e-20
Ct21-4487	CAQ16270	Hypothetical protein	*Glomerella graminicola*	7e-18
Contig 9	XP_001557501	Predicted protein	*Botryotinia fuckeliana*	9e-09
Ct21-2424	CAA04765	Glycoprotein CIH1	*Glomerella lindemuthiana*	2e-27
Ct21-2435	CAA04765	Glycoprotein CIH1	*Glomerella lindemuthiana*	2e-22
Ct21-3485	CAA04765	Glycoprotein CIH1	*Glomerella lindemuthiana*	8e-29
Ct21-1783	XP_960686	Clock-controlled protein 6	*Neurospora crassa*	1e-12
Ct21-4525	XP_960686	Clock-controlled protein 6	*Neurospora crassa*	1e-11
Contig 29	XP_960686	Clock-controlled protein 6	*Neurospora crassa*	3e-11
Ct21-3233	XP_002340703	Extracellular conserved serine-rich protein	*Talaromyces stipitatus*	1e-19
Contig 11	XP_746687	Conserved glycine-rich protein	*Aspergillus fumigatus*	3e-22
Ct21-160	XP_775248	Hypothetical protein	*Glomerella graminicola*	9e-08
Ct21-3817	XP_365520	Hypothetical protein	*Magnaporthe grisea*	1e-50
Ct21-3527	XP_001910524	Hypothetical protein	*Podospora anserina*	2e-05
Contig 28	XP_001264578	MFS sugar transporter	*Neosartorya fischeri*	6e-87
Contig 30	XP_389647	Glucose-regulated protein homolog precursor GRP 78	*Gibberella zeae*	0.0*
Ct21-2300	AAM00019	ToxB precursor	*Pyrenophora tritici-repentis*	3e-04
Ct21-3042	EEY18399	Cu-Zn superoxide dismutase	*Verticillium albo-atrum*	1e-45
Ct21-3890	EEY18083	Peptidyl-prolyl cis-trans isomerase B	*Verticillium albo-atrum*	3e-81
Ct21-4099	EEY20068	Chitin deacetylase	*Verticillium albo-atrum*	8e-83
Ct21-4275	XP_001831864	Metalloreductase	*Coprinopsis cinerea*	4e-14
Ct21-1979	EEY19380	Integral membrane protein	*Verticillium albo-atrum*	3e-43
Contig 12	XP_001553407	Predicted protein	*Botryotinia fuckeliana*	0.015*
Ct21-43	EEY14873	Conserved hypothetical protein	*Verticillium albo-atrum*	6e-69
Ct21-2181	XP_001906422	Hypothetical protein	*Podospora anserina*	1e-23
Ct21-1105	XP_387661	Hypothetical protein	*Gibberella zeae*	9e-39
Ct21-2709	XP_387661	Hypothetical protein	*Gibberella zeae*	1e-35
Ct21-3808	XP_364081	Hypothetical protein	*Magnaporthe grisea*	9e-21
Ct21-4329	XP_001592593	Hypothetical protein	*Sclerotinia sclerotiorum*	5e-18
***Candidate effectors***				
Contig 6	EEY19659	Conserved hypothetical protein	*Verticillium albo-atrum*	7e-29
Contig 8	ABE73692	Eliciting plant response-like protein	*Hypocrea atroviridis*	2e-45
Contig 10	XP_386615	Hypothetical protein	*Gibberella zeae*	2e-80
Contig 31	EEU39436	Small secreted protein	*Nectria haematococca*	1e-07
Ct21-741	EEY14856	Conserved hypothetical protein	*Verticillium albo-atrum*	9e-36
Ct21-1631	EEY16152	Conserved hypothetical protein	*Verticillium albo-atrum*	5e-59
Contig 32	ABK76310	Hypersensitive response-inducing protein	*Ceratocystis ulmi*	7e-23
Ct21-2867	EEU38174	Hypothetical protein	*Nectria haematococca*	4e-36
Ct21-4630	EEU46355	Predicted protein	*Nectria haematococca*	1e-45
Contig 5	ACF19427	Extracellular protein 6	*Passalora fulva*	0.028*
Ct21-1573	ACF19427	Extracellular protein 6	*Passalora fulva*	1e-22
***Hydrolases***				
Contig 13	EEY15172	Aminopeptidase Y precursor, putative	*Verticillium albo-atrum*	5e-75
Contig 14	XP_956796	Carboxypeptidase Y precursor	*Neurospora crassa*	9e-108
Contig 15	ACM42424	Subtilisin serine protease	*Chaetomium globosum*	1e-61
Ct21-125	B8XGR2	Carboxypeptidase 2	*Trichophyton equinum*	4e-19
Ct21-572	EEY23108	Serin endopeptidase	*Verticillium albo-atrum*	1e-42
Ct21-708	AAS45251	Subtilisin-like serine protease	*Verticillium dahliae*	4e-27
Ct21-1114	XP_002843648	Glutamate carboxypeptidase 2	*Arthroderma otae*	5e-110
Ct21-1766	EEY22919	Peptidase M14	*Verticillium albo-atrum*	4e-99
Ct21-1999	XP_001940792	Carboxypeptidase Y precursor	*Pyrenophora tritici-repentis*	5e-44
Ct21-2223	EEY15034	Metalloprotease	*Verticillium albo-atrum*	1e-49
Ct21-2830	ACV96842	Aspartic proteinase	*Botryotinia fuckeliana*	4e-63
Ct21-3428	CAL25580	Serin endopeptidase	*Hypocrea lixii*	2e-72
Ct21-3969	CAB44651	Chymotrypsin	*Metarhizium anisopliae*	3e-62
Ct21-4220	EEY23006	Conserved hypothetical protein	*Verticillium albo-atrum*	2e-88
Ct21-4840	EEY23418	Serin endopeptidase	*Verticillium albo-atrum*	2e-22
Ct21-4964	EEY21352	Aminopeptidase Y	*Verticillium albo-atrum*	1e-52
Contig 16	EDP48344	Pectin lyase B	*Aspergillus fumigatus*	5e-75
Contig 17	XP_001935104	Cutinase precursor	*Pyrenophora tritici-repentis*	6e-60
Contig 18	O94218	Xyloglucan-specific endo-beta-1,4-glucanase precursor	*Aspergillus aculeatus*	5e-37
Contig 19	ABC65824	Cell wall glucanosyltransferase	*Metarhizium anisopliae*	3e-65
Contig 20	EEY20211	Cutinase-2	*Verticillium albo-atrum*	3e-38
Contig 21	XP_002382421	Esterase, putative	*Aspergillus flavus*	2e-26
Ct21-1152	XP_002373471	Lipase/esterase, putative	*Aspergillus flavus*	9e-25
Ct21-1372	XP_002379112	Esterase, putative	*Aspergillus flavus*	6e-46
Ct21-2524	EEY22945	Hydrolase (Esterase/Lipase domain)	*Verticillium albo-atrum*	3e-27
Ct21-2039	EEY15257	Pectinesterase	*Verticillium albo-atrum*	1e-78
Contig 25	CAC29255	Pectin methyl esterase	*Botryotinia fuckeliana*	1e-14
Contig 22	EEY23761	Pectate lyase	*Pyrenophora tritici-repentis*	6e-79
Contig 23	XP_001933274	Endoglucanase II	*Pyrenophora tritici-repentis*	5e-61
Contig 24	XP_001940096	Glycosyl hydrolase	*Pyrenophora tritici-repentis*	5e-42
Contig 26	EEU40344	Glycoside hydrolase family 43	*Nectria haematococca*	2e-117
Contig 27	XP_001261592	Pectate lyase, putative	*Neosartorya fischeri*	3e-39
Ct21-435	EEY22330	Cell wall glycosyl hydrolase	*Verticillium albo-atrum*	2e-137
Ct21-551	XP_001940634	Periplasmic beta-glucosidase precursor	*Pyrenophora tritici-repentis*	2e-98
Ct21-605	EEY19750	Septation protein SUN4	*Verticillium albo-atrum*	3e-42
Ct21-657	EEU42734	Glycoside hydrolase family 76	*Nectria haematococca*	7e-46
Ct21-658	XP_001939866	Arabinosidase	*Pyrenophora tritici-repentis*	3e-177
Ct21-817	EEY20388	Cell wall glycosyl hydrolase	*Verticillium albo-atrum*	2e-120
Ct21-1102	NP_592836	Cell wall protein Asl1, O-glucosyl hydrolase	*Schizosaccharomyces pombe*	1e-12
Ct21-1170	CAC14022	Endopolygalacturonase	*Colletotrichum gloeosporioides *f. sp. *malvae*	1e-78
Ct21-1507	ABC65824	Cell wall glucanosyltransferase	*Metarhizium anisopliae*	8e-56
Ct21-1571	EEY17994	Beta-glucanase	*Verticillium albo-atrum*	3e-109
Ct21-1584	AAQ23181	Extracellular lipase	*Gibberella zeae*	5e-66
Ct21-1644	EEY23761	Pectate lyase	*Verticillium albo-atrum*	3e-50
Ct21-1737	EEY19876	Beta-glucosidase	*Verticillium albo-atrum*	1e-3
Ct21-1738	EEY23761	Pectate lyase	*Verticillium albo-atrum*	3e-50
Ct21-1739	XP_001937817	Fungal cellulose binding domain containing protein	*Pyrenophora tritici-repentis*	3e-34
Ct21-3571	XP_001937817	Fungal cellulose binding domain containing protein	*Pyrenophora tritici-repentis*	9e-35
Ct21-4548	XP_001937817	Fungal cellulose binding domain containing protein	*Pyrenophora tritici-repentis*	3e-40
Ct21-1786	EEY20352	Phosphoserine phosphatase	*Verticillium albo-atrum*	2e-112
Ct21-1816	BAH10648	Beta-L-arabinopyranosidase/alfa-D-galactopyranosidase	*Fusarium oxysporum*	2e-81
Ct21-1965	EEU44929	Glycoside hydrolase family 12	*Nectria haematococca*	4e-40
Ct21-1989	ACZ06599	Exopolygalacturonase	*Fusarium oxysporum f. sp. lycopersici*	1e-96
Ct21-2762	XP_001216041	Endoglucanase I precursor	*Aspergillus terreus*	7e-09
Ct21-2876	XP_958254	Endoglucanase IV precursor	*Neurospora crassa*	5e-56
Ct21-3095	EEY21635	Mannan endo-1,4-beta-mannosidase	*Verticillium albo-atrum*	2e-103
Ct21-3342	EEY20111	Glycosyl hydrolase family 43 protein	*Verticillium albo-atrum*	5e-119
Ct21-3508	AAM77705	Endoglucanase	*Nectria ipomoeae*	6e-66
Ct21-3569	ABH03046	Pectin lyase I	*Penicillium occitanis*	4e-63
Ct21-3767	XP_001259883	Extracellular cellulase CelA, putative	*Neosartorya fischeri*	3e-54
Ct21-3948	EEY21541	Alpha-galactosidase	*Verticillium albo-atrum*	1e-115
Ct21-4357	XP_001937210	Beta-1,6-galactanase	*Pyrenophora tritici-repentis*	1e-17
Ct21-4443	XP_001836535	Endoglucanase B	*Coprinopsis cinerea*	6e-43
***Other proteins***				
Ct21-153	AAL08969	Glycine-rich protein	*Coccidioides posadasii*	1e-27
Ct21-1373	EFQ36857	NUDIX-domain containing protein	*Glomerella graminicola*	1e-46
Ct21-1408	CAD71220	Chloroperoxidase related protein	*Neurospora crassa*	1e-46
Ct21-3930	EEH44533	Glutaminase	*Paracoccidioides brasiliensis*	6e-62
Ct21-4401	EEY18112	L-ascorbate peroxidase	*Verticillium albo-atrum*	4e-06
Ct21-4758	EEY17786	Predicted protein (NAD(P)(+)-binding protein region)	*Verticillium albo-atrum*	0.006*
Ct21-3336	XP_360331	Predicted protein	*Magnaporthe grisea*	6e-05
Ct21-25	EEY16153	Conserved hypothetical protein	*Verticillium albo-atrum*	3e-60
Ct21-695	EEU39144	Hypothetical protein	*Nectria haematococca*	2e-21
Ct21-1015	EEU42792	Hypothetical protein	*Nectria haematococca*	4e-23
Ct21-1283	CAQ16193	Hypothetical protein	*Glomerella graminicola*	2e-05

* Unigenes with *E *value > 10^-5 ^are classified according to their conserved signatures as described in the text.

### Hydrolytic enzymes

Sixty-three unique sequences showed significant similarity to members of the enzyme classes (EC) 3 (hydrolases) and 4 (lyases). Most of them had been annotated as cell wall degrading enzymes (CWDEs). Ten unigenes encoded enzymes acting on ester bonds (EC 3 subclass 3.1); most of them were reported as pectinolytic enzymes (pectinases). These included esterases, pectinesterase, extracellular lipase, phosphoserine phosphatase and cutinase. Four depolymerase pectinolytic enzymes (polygalacturonases [EC 3 subclass 3.2] and lyases [EC 4 subclass 4.2]) were also retrieved from this library. Unigenes Ct21-1170 and Ct21-1989 were similar to exopolygalacturonase and endo-polygalacturonase, respectively. Four discrete unigenes contig 22, Ct21-1644, Ct21-1738 and Ct21-3569 encoded pectate lyase (polygalacturonate lyase) and contig 16 showed homology to pectin lyase (polymethylgalacturonate lyase).

Thirty unique sequences, including Ct21-1170 and Ct21-1989 mentioned above represented the EC 3 subclass 3.2 glycosidases (glycosyl hydrolases). Glycosidases hydrolyze the bonds between carbohydrates and release sugars from plant cell walls to provide the fungus with carbon sources and support host penetration and colonization [19]. These included xyloglucan-specific endo-β-1, 4-glucanase, cell wall glucanosyltransferase, cellulase (endoglucanases of class I, II and IV, and extracellular cellulase CelA), glycosyl hydrolase family proteins, β-glucosidase, arabinosidase, β-glucanase, mannan endo-1,4-β-mannosidase and α-galactosidase.

Sixteen unigenes belonged to the EC 3 subclass 3.4 of enzymes acting on peptide bonds (peptidases; aminopeptidase Y, serine type carboxypeptidase, glutamate carboxypeptidase II, carboxypeptidase A, serine endopeptidase, subtilisin serine protease, chymotrypsin, aspartic proteinase and metalloprotease). Correlation between peptidase activity and virulence for several phytopathogenic fungi has already been established.

### Cell envelope-associated proteins (CEAPs)

Forty-three unigenes carrying SP, transmembrane helices or GPI anchor addition signal, including 14 unigenes homologous to functionally uncharacterized genes from fungi, were identified as CEAPs. Among them, six unigenes were classified under the hydrolytic enzymes category. In almost all cases except the unigene Ct21-160, the best match for these unigenes was found within the Ascomycota. Interestingly, most of the identified genes have not been previously reported in the genus *Colletotrichum*.

C-terminal GPI anchor sites were predicted in the translated amino acid sequences of 11 unigenes. Interestingly, we identified a member of the GPI transamidase complex (Ct21-1020) that is implicated in en bloc synthesis of GPI proteins. Three unigenes contig 1, contig 2 and Ct21-4350 encoded GPI anchored serine-threonine rich proteins. Two unigenes, contig 7 and Ct21-3268, were predicted to possess GPI anchor addition signals and cysteine rich fungal extracellular membrane (CFEM) domains (pfam05730). Unigene Ct21-4487 was identified as a hypothetical protein that possesses a GPI addition signal. Contigs 3 and 4 were matched to a putative GPI-anchored protein and GPI-anchored serine rich protein, respectively. Clones Ct21-949 and Ct21-1283 were matched to the GPI anchored cell wall organization protein Ecm33 from *Pyrenophora tritici-repentis *and glycolipid anchored surface protein GAS1 from *C. graminicola*, respectively.

The predicted products of the three discrete unigenes Ct21-2424, Ct21-2435 and Ct21-3485 resembled the biotrophy associated secreted glycoprotein *Colletotrichum *intracellular hypha 1 (CIH1) from *C. lindemuthianum*, the causal agent of common bean anthracnose [20]. The ORF of Ct21-2435 encoded a protein of 168 amino acids containing 2 lysin motif (lysM) domains. These lysMs recognize and bind N-acetyl D-glucosamine [21], and may play a role protecting fungal chitin from plant chitinases. The predicted ORFs from three discrete clones, contig 29, Ct21-1783 and Ct21-4525 encoded a protein that was similar to clock controlled protein 6 from *Neurospora crassa*, induced by light and during conidial development [22].

Contig 28 encoded a major facilitator superfamily (MFS) of sugar transporter that contained six transmembrane domains. MFS transporters are involved in the symport, antiport and uniport of various substrates, such as sugars, Krebs cycle intermediates, phosphate esters, oligosaccharides and antibiotics [23]. Contig 30 was similar to glucose-regulated protein 78 (GRP78) in that it is not an extracellular protein. GRP78 is a subgroup of heat shock protein 70 and located in the ER lumen where it assists in the post-translational import and folding of proteins [24] as well as the reversible binding of misfolded and underglycosylated proteins [25]. Clone Ct21-2300 was matched to a toxb precursor of *P. tritici-repentis*. Toxb is a host specific chlorosis toxin and was identified as a potential pathogenicity factor in *P. tritici-repentis*, the causal agent of tan spot of wheat [26].

Several enzymes were found to possess transmembrane domains and hence were classified as CEAPs. The Cu/Zn superoxide dismutase (Ct21-3042) is a key enzyme in the dismutation of superoxide radicals resulting from cellular oxidative metabolism into hydrogen peroxide [27]. Clone Ct21-3890 matched an enzyme called peptidyl-prolyl cis-trans isomerase B (PPIase) (EC 5.2.1.8). PPIase possesses a chaperone activity to fold proteins into active configuration by catalyzing *cis/trans *isomerization on proline-peptide bonds [28]. An enzyme chitin deacetylase (Ct21-4099) involved in modifying fungal cell wall was also identified. Two unigenes, Ct21-4275 and Ct21-4401, encoded metalloreductase and ascorbate peroxidase oxidoreductases, respectively. The metalloreductase contained a transmembrane domain, hence was classified as CEAP, whereas the ascorbate peroxidase lacked a transmembrane domain, and was therefore included under the category of other proteins.

### Candidate effector proteins

It is assumed that fungal effectors are likely to be small [< 300 amino acids (aa)], cysteine-rich and soluble extracellular proteins that do not become cross linked into fungal cell walls [13]. With the exponential rise in the number of fungal phytopathogen genomes being sequenced, the likelihood of finding effector orthologs in other species is increasing. For example, *C. fulvum *lysM effector ECP6 has orthologs from 11 filamentous fungal species, including 7 plant pathogens [21]. We identified 11 secreted proteins (encoded by contigs 5, 6, 8, 10, 31, 32, Ct21-741, Ct21-1573, Ct21-1631, Ct21-2867, and Ct21-4630) that met characteristic criteria, including 8 proteins homologous to functionally uncharacterized proteins from fungal species. The predicted proteins encoded by these unigenes shared the characteristics of being small (112 to 296-aa), lacking a transmembrane domain, and being relatively rich in cysteine (3 to 16 residues). The functionally annotated homologs to two additional clones (contig 29 and Ct21-1783) also shared these features. These candidate effector proteins were predicted to possess cysteine disulfide bridges that may aid protein stability in the extracellular milieu and inside the host cell to protect them from degradation [29,30]. Putative disulfide bond spacing was conserved between predicted ORFs and similar proteins available in the public domain. Among the functionally annotated proteins, two discrete unigenes (contig 5 and Ct21-1573) were similar to *C. fulvum *lysM effector protein ECP6 induced during the colonization of tomato [21]. However, unlike ECP6, contig5 and Ct21-1573 contained 1 and 2 lysM, respectively. Contig 8 exhibited similarity to an eliciting plant response-like protein that contained a CP domain. CP proteins from *Ceratocystis fimbriata *are surface proteins that induce necrosis and elicit phytoalexin synthesis in plants [31]. A unigene (contig 31) was matched to a small secreted protein (139-aa) from *Nectria haematococca *with 7 cysteine residues (form 3 disulfide bonds) in the mature protein. Contig 32 was similar to a HR-inducing protein from *Ceratocystis ulmi*. Homologs to 7 unigenes were hypothetical proteins.

### Other proteins

The predicted peptides of a group of 11 unigenes showed similarities to diverse proteins from fungal genome sequences. The encoded peptide of clone Ct21-1408 was similar to a chloroperoxidase related protein. Chloroperoxidase catalyzes the insertion of chlorine, bromine and iodine atoms into organic acceptor molecules. EST clone Ct21-3930 was aligned with glutaminase, an enzyme catalyzing the hydrolysis of glutamine to glutamic acid. Glutaminases play a role in the acquisition of nitrogen from less preferred sources [32]. The oxidoreductase enzyme L-ascorbate peroxidase catalyzes the chemical reaction L-ascorbate + H_2_O_2 _↔dehydroascorbate + 2 H_2_O. The remaining 7 unigenes could not be classified by homology search into known or putative proteins (Table 1). However, sequence analyses of predicted peptides revealed that most of these unigenes were rich in glycine or leucine. Ct21-1373 was identified as a nudix/MutT family protein nudix hydrolase (*Pyrenophora tritici-repentis*). Nudix hydrolases hydrolyze substrates of general structure nucleoside diphosphate linked to another moiety, X to yield nucleotide monophosphate and P-X [33].

### Time-course expression analysis of genes encoding putative effector proteins and CEAPs

Using semiquantitative RT-PCR, we examined transcript levels of genes encoding candidate effectors and CEAPs (GPI-proteins and CIH1) identified from the EST library in different cell types formed *in vitro*, viz. ungerminated conidia, mycelia and mycelia grown in minimal media supplemented with lentil leaflet cell walls (referred to hereafter as cell wall treated mycelia). Expression was also analyzed *in planta *at various stages of plant infection, namely appressorial penetration (16 hai), primary hyphae (44 hai) and secondary hyphae (68 hai) (Figure 2B).

**Figure 3:**
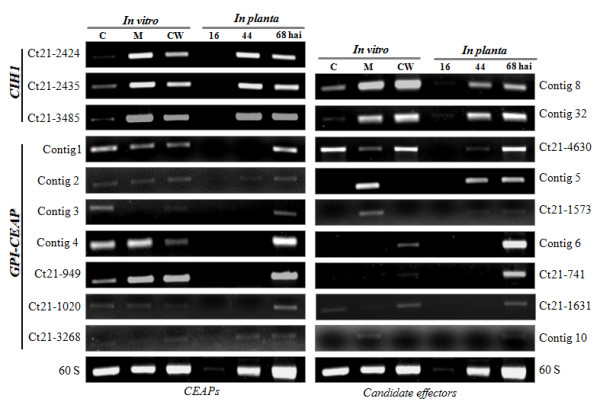
**Transcript abundance dynamics of CEAPs and candidate effectors.** Expression profiling of transcripts encoding GPI-anchored proteins, CIH1 and candidate effectors by semiquantitative RT-PCR. C, conidia; M, mycelia; and CW, cell wall treated mycelia of *C. truncatum*.

Time-course expression profiles were obtained for 9 candidate effector encoding genes (Figure 3). When considering the *in planta *infection process, two kinds of expression patterns could be distinguished. EST clones contig 5, 8, 32, Ct21-1573, and Ct21-4630 were expressed at both *in planta *biotrophic- and necrotrophic-developmental stages. Expression at lower level was also detected in the appressorial penetration stage for two clones, contigs 8 and 32. Three clones, contigs 8, 32, and Ct21-4630 were expressed constitutively in all *in vitro *cell types. Clones, contig 5 and Ct21-1573 identified as homologs of lysM effector Ecp6 [21], were expressed in mycelia, whereas they were undetectable in conidia and cell wall treated mycelia, suggesting the possible involvement of these effectors genes in vegetative growth. Transcripts from four genes (contigs 6, 10, Ct21-741, and Ct21-1631) were exclusively accumulated during the necrotrophic phase of the infection process. Among them, two genes (contig 6 and Ct21-741) also showed expression in the cell wall treated mycelia, however, at a lower level than during the *in planta *necrotrophic phase, whereas contig 10 appeared to be expressed in mycelia and cell wall treated mycelia. Clone Ct21-1631 showed constitutive expression in all cell types tested.

Figure 3 shows expression profiles of seven (contigs 1, 2, 3, 4, Ct21-949, Ct21-1020 and Ct21-3268) out of eleven genes encoding GPI-CEAPs. All genes showed constitutive varying expression pattern in all *in vitro *cell types. In addition, they all were undetectable during appressorial penetration except for Ct21-3268, and during the biotrophic phase except for Contig 2 and Ct21-3268, but all were abruptly expressed during the necrotrophic phase. This was well corroborated by the abundance dynamics of the gene encoding GPI transamidase (Ct21-1020), an enzyme involved in the biosynthesis of GPI-CEAPs that was completely shut down in the biotrophic phase. These data suggest that GPI-CEAPs accumulated during the *in planta *necrotrophic phase and *in vitro *growth.

Another subclass of CEAPS tested for expression was CIH1 protein encoding unigenes (Ct21-2424, Ct21-2425 and Ct21-3485). All three were constitutively expressed in all cell types tested, indicating their role in vegetative development of the pathogen. In addition, their transcripts were also accumulated at both biotrophic- and necrotrophic- *in planta *developmental stages, suggesting that CIH1 glycoprotein is a basal component of the cell wall of *C. truncatum *as described previously [20].

### Functional analysis of selected candidate effectors by PVX agroinfiltration assay

To investigate if candidate effectors have elicitor activity, we expressed 3 candidate effector proteins (encoded by unique sequences contigs 6, 8 and 32) and *M. oryzae *CP protein by mean of agroinfiltration in tobacco leaves.

EST analysis of contig 6 revealed a cDNA of 784-bp with an ORF of 417-bp encoding a 139-aa preprotein. A SP of 15-aa with a cleavage site in between glycine-15 and serine-16 was predicted in the preprotein, and 7 cysteine residues were found in the mature protein (124-aa). Using DISULFIND server, three cystine disulfide bridges were predicted in the mature protein at a confidence level of > 0.5. This protein showed no homology to any functionally characterized protein. The cDNA of contig 8 was 775-bp in size with an ORF of 414-bp encoding a 138-aa preprotein. A SP of 21-aa with a cleavage site between valine-21 and serine-22, and a CP domain spanned from 23-aa to 138-aa were predicted at the N- and C-termini, respectively. The CP domain contains 4 cysteine residues, which are known to form two disulphide bonds [31]. Proteins belonging to the CP family share common characteristics, such as a small size, an N-terminal SP for secretion and a conserved cysteine residue pattern [34,35]. Protein encoded by unigene contig 8 possessed all features of a CP protein, hence we termed it CtCP1. Sequence alignment of CtCP1 with homologues from other fungal species revealed that CtCP1 showed significant similarity with fungal proteins of CP family like *M. oryzae *SM1 (MGG_05344), *Hypocrea atroviridis *eliciting plant response like protein 1 (Epl; DQ464903), *Hypocrea virens *snodprot1 (DQ494198) and *Gibberella pulicaris *snodprot-FS (AY826795) (Additional file 3). Based on sequence features, CtCP1 belongs to Epl1 cluster of CP proteins [35]. Recently, a *M. oryzae *CP protein MgSM1 was shown to induce HR and confer broad-spectrum disease resistance in *Arabidopsis *[36]. EST analysis of contig 32 revealed a cDNA of 686-bp with an ORF of 426-bp encoding a 142-aa preprotein. A SP of 17-aa with a cleavage site between alanine-17 and isolecine-18 was predicted at the N-terminus of the preprotein, and 4 cysteine residues were predicted in the mature protein (125-aa). This protein showed significant homology to the HR inducing protein of *Ceratocystis ulmi *and contained 4-cysteine residues.

**Figure 4:**
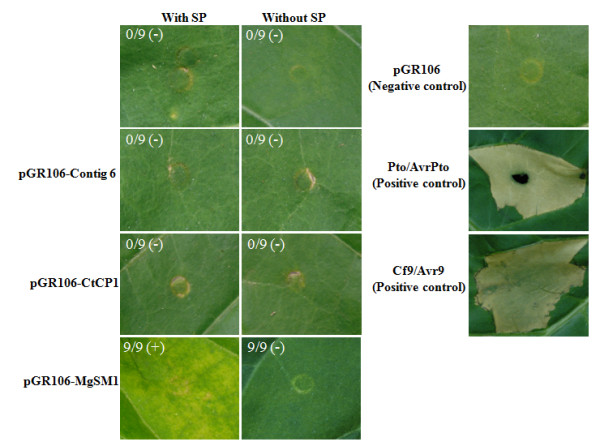
***In planta*****expression of Contigs 6, 32, CtCP1 and MgSM1***. In planta *expression of Contigs 6, 32, CtCP1 and MgSM1 (A) *N. tobacum *leaves were challenged by agro-infiltration of *A. tumefaciens *strains expressing pGR106-contig 32^1-142 ^(SP), pGR106-contig 32^16-142 ^(-SP), pGR106-contig 6^1-139 ^(SP), pGR106-contig 6^16-139 ^(-SP), pGR106-CtCP1^1-138 ^(SP), pGR106-CtCP1^22-138 ^(-SP), pGR106-MgSM1^1-137 ^(SP) and pGR106-MgSM1^22-137 ^(-SP). Empty vector pGR106 and matching pairs of R/Avr (Pto/AvrPto and Cf9/Avr9) were used as negative and positive controls, respectively. Pictures were taken 7 days after infiltration. SP, Signal peptide.

**Figure 5:**
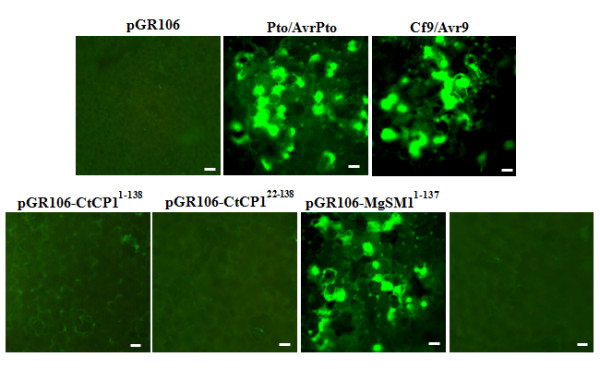
**Detection of polyphenolic compound accumulation.** UV-stimulated autofluorescence of tobacco leaf tissues collected from the edges of pGR106, R/Avr pairs (Pto/AvrPto and Cf9/Avr9), pGR106-CtCP1^1-138^, pGR106-CtCP1^22-138 ^and pGR106-MgSM1^1-137 ^and pGR106-MgSM1^22-137 ^infiltrated zones. Bars = 20 μm.

The ORFs of three candidate effectors from *C. truncatum *and MgSM1 from *M. oryzae *with and without SP were cloned into PVX vector pGR106 [pGR106-contig 6^1-139^, pGR106-contig 6^16-139^, pGR106-CtCP1^1-138^, pGR106-CtCP1^22-138^, pGR106-contig 32^1-142^, pGR106-contig 32^16-142^, pGR106-MgSM1^1-137 ^and pGR106-MgSM1^22-137^] and transformed into *Agrobacterium tumefaciens *strain GV3101. We used the empty vector pGR106 as negative control, which upon transient expression showed no micro- and/or macroscopic phenotype. We also incorporated two R/Avr protein pairs as positive controls that have been shown to interact and trigger cell death in tobacco: Pto/AvrPto [37] and Cf9/Avr9 [38]. Agroinfiltration of recombinant *A. tumefaciens *strains carrying pGR106-MgSM1^1-137 ^in tobacco leaves resulted in macroscopic cell death 5 days after infiltration, whereas the rest of the strains were unable to cause any macroscopic phenotype (Figure 4). Transient co-expression of the genes that encode Avr/R protein pairs (Pto/AvrPto and Cf9/Avr9) elicited a spotted faint glazing on the tobacco leaf surface 19 h post infiltration, which progressed to tissue collapse and became confluent 5 days post infiltration. To investigate whether CtCP1 protein causes any microscopic cell death, we collected leaf tissues from the edges of pGR106, pGR106-CtCP1^1-138^, pGR106-CtCP1^22-138^, pGR106-MgSM1^1-137 ^and pGR106-MgSM1^22-137 ^infiltration zones for polyphenolic compound- associated autofluorescence under ultraviolet (UV) light. The pGR106, pGR106-CtCP1^1-138^, pGR106-CtCP1^22-138 ^and pGR106-MgSM1^22-137 ^edges had no autofluorescence under UV light, whereas pGR106-MgSM1 edges showed strong autofluorescence under UV light (Figure 5).

## Discussion

The morphological and nutritional transition of a hemibiotrophic pathogen from biotrophy to necrotrophy plays a critical role in disease development. During this switch, fungal phytopathogens may secrete large repertoires of secretory proteins to manipulate host innate immunity in concert with a destructive arsenal of CWDEs that manifest the disease phenotype. Our strategy in this study was to catalogue genes encoding secretory proteins from *C. truncatum *by constructing a directional cDNA library representing the biotrophy-necrotrophy switch and then to exploit biocomputational tools to scan resulting fungal ESTs for proteins containing canonical N-terminal SPs. To date, cDNA library- and EST-based studies have been conducted to identify secretory protein encoding genes from haustoria of *Puccinia striiformis *f.sp. *tritici *[39], *C. higginsianum *appressoria [13], *in planta *infection stages of *Venturia inaequalis *[14] and mycelia of the corn pathogen *C. graminicola *grown *in vitro *under different secretion inducing conditions [15]. There has been no previous EST-based analysis of the biotrophy-necrotrophy switch for any fungal pathogen. Our library contained 122 fungal unigenes encoding secretory proteins. Functional classification of the secretory proteins based on homology searches and sequence analyses revealed the presence of four potential groups: hydrolytic enzymes, CEAPs, candidate effector proteins and other proteins.

Hemibiotrophs orchestrate a physiological switch from asymptomatic infection to massive cell death and tissue dissolution, presumably resulting from the coordinated secretion of cell-death elicitors and hydrolytic enzymes [40]. With 63 of 122 unigenes belonging to EC classes 3 and 4, hydrolases and lyases are a major group of enzymes identified at the biotrophy-necrotrophy switch. In general, fungal pathogens secrete a battery of CWDEs capable of hydrolyzing host cell wall components (cellulose, hemicellulose, pectin and structural proteins) to accommodate a necrotrophic life style. These CWDEs include carbohydrate esterases (pectinases), glycosyl hydrolases, polysaccharide lyases (pectinases) and peptidases, which the fungal pathogens utilize to degrade the host plant cell wall for nutrition [41]. The glycosidases like galactosidase and arabinosidase may liberate sugar moieties that can be used as a nutritional source for *C. truncatum *during the necrotrophic growth. Glycosidases may also have a role in decreasing the steric hinderance for other CWDEs by removing protruding side chains from the backbone of polymers and providing increased access for endo forms of CWDEs [42]. Pectinases weaken the cell wall and expose cell wall components to other enzymes, such as cellulases and hemicellulases. Genome sequencing has revealed that the hemibiotrophic phytopathogen *M. oryzae *is predicted to possess 121 glycosyl hydrolases, 4 polysaccharide lyases, and 13 carbohydrate esterases [41]. Our library retrieved 15 glycosyl hydrolases, 2 polysaccharide lyases, and 5 carbohydrate esterases. Another class of identified hydrolases was peptidases that may have a function in the extracellular processing of fungal secreted proteins [43], degradation of proteinaceous components involved in the plant response against pathogens [44] or degradation of host tissue [45]. Esterases, glycosyl hydrolases, lyases, and peptidases could also weaken host cell walls, allowing for an easier access to nutrients.

The interaction between a pathogen and its host is to a large extent orchestrated by the proteins that are secreted or localized to or at the cell membrane or cell wall (CEAPs). The CEAPs are secreted through the endoplasmic reticulum (ER) pathway and tethered either to the fungal cell membrane or wall. Some CEAPs lack a transmembrane domain but possess C-terminal GPI anchor addition signal. GPI anchoring is an important mechanism to tether extracellular proteins to the plasma membrane or cell wall [46]. The carboxyl termini of these proteins have a sequence motif that is recognized by a protein complex located in the ER, known as the GPI transamidase (unigene Ct21-1020). The GPI transamidase complex cleaves the protein at a position within this motif, termed the omega site, and transfers the GPI anchor en bloc to the newly generated C terminus of the protein. A genome-wide inventory of secretory proteins of *P. sojae *and *P. ramorum *revealed putative secretomes of 1,464 and 1,188 proteins, respectively [47], of which a subset of approximately 100 proteins contain putative GPI anchor addition signals. The majority of elicitins (capable of inducing an HR in tobacco) from *Phytophthora *possess a GPI anchor [48]. Components of the cell envelope of pathogens can be rapidly acclimatized by a changing environment. The ability to respond to a changing milieu is critical for pathogens to effectively evade, tolerate and/or suppress host immune responses. Therefore, the structure and composition of the cell envelope is dynamic and changes during different stages of *in planta *growth. We observed an abrogation or a reduction in transcript levels of genes encoding CEAPs during the biotrophic phase and an abrupt induction in the necrotrophic phase (Figure 3). The switch from voluminous biotrophic hyphae to thin necrotrophic hyphae involves an increased surface area to volume ratio, therefore may require elevated transcription of some of these cell surface protein encoding unigenes. This ratio and thinner cell walls of the secondary hyphae would be advantageous for both efficient nutrient uptake and secretion of wall degrading enzymes, and possibly toxins, during the necrotrophic growth of hemibiotrophic fungal pathogens [49].

The predicted products of three discrete unigenes Ct21-2424, Ct21-2435 and Ct21-3485 resembled the biotrophy associated protein CIH1 from *C. lindemuthianum *[24]. The CIH1 was shown to be secreted into the cell wall of invasive biotrophic hyphae and the plant-pathogen interface. In the present study, we found that the *C. truncatum *CIH1 homolog was expressed constitutively in all *in vitro *cell types tested and both during the biotrophic- and necrotrophic- phases. This result was contradictory to studies with *C. higginsianum *where the CIH1 protein was specifically expressed in the biotrophic phase [16]. It suggests either the transcription level of *CIH1 *does not reflect the protein level, or *C. truncatum *may employ a different approach to host invasion that is unlike that of other species of *Colletotrichum*.

Based on the *in planta *transcript profiling of nine genes encoding putative effector proteins, we identified two types of expression patterns: biotrophic/necrotrophic-specific and necrotrophic-specific. Transient expression of three candidate effectors in tobacco leaves revealed that none of them was able to elicit an HR response. Surprisingly, our data on CtCP1 were in contrast of that recently published on *M. oryzae *CP protein, MgSM1. MgSM1, upon transient expression, stimulated HR and conferred broad-spectrum resistance to Arabidopsis against *Botrytis cinerea *and *Pseudomonas syringae *pv. *tomato *[40].

The predicted products of two discrete unigenes (contig 5 and Ct21-1573) showed homology to extracellular protein 6 (ECP6) from *C. fulvum*. LysMs are N-acetyl D-glucosamine (chitin) binding modules that have been found in mammalian and plant pathogenic fungi as well as in saprophytes [50]. Therefore, they may also have a role in counter defense by protecting fungal cell walls against plant chitinases. Bolton et al. [21] demonstrated that ECP6 was induced by the plant pathogenic fungus *C. fulvum *during the initial infection of tomato and acted as a virulence factor. During infection, LysM domain-containing protein Ecp6 sequesters chitin oligosaccharides that are released from the cell walls of invading hyphae to avert the elicitation of host immunity. This sequestering may represent a common strategy of host immune suppression by fungal pathogens, because LysM effectors are widely conserved in the fungal kingdom [51].

We identified several ESTs that may encode novel effectors. Using bioinformatics tools, we identified 5 soluble secreted proteins of unknown function (contig 10, Ct21-741, Ct21-1631, Ct21-2867 and Ct21-4630). Three of them (contig 10, Ct21-741, Ct21-1631) were not expressed in the biotrophic phase while induced during the necrotrophic phase. Their expression was also detected either in some or all of the *in vitro *cell types. Such genes may be important for establishing fungal necrotrophy. One (Ct21-4630) was constitutively expressed during all *in planta *developmental stages except appressorial penetration and in all *in vitro *cell types (Figure 3), suggesting its general role in both vegetative development and pathogenesis.

## Conclusions

We catalogued proteins putatively secreted by *C. truncatum *and several candidate effectors had been mined from the EST library. It was observed that the transcript levels of candidate effector genes were either induced during the necrotrophic growth of the pathogen compared with that during the biotrophic growth or elevated during both phases. Through *in planta *expression of candidate effectors, we demonstrated the functional role of one effector. In summary, our study suggests that suppression of host defense during the biotrophic phase and the subsequent unmasking of PAMPs or elicitors during the necrotrophic phase may be involved in pathogenesis of *C. truncatum*.

## Methods

### Plant material and cell wall extraction

Lentil plants of the Canadian cultivar 'Eston', which is fully susceptible to *C. truncatum *isolate CT-21, were grown at four plants per 18 cm diameter pot filled with a commercial potting soil mixture (Terralite Redi-Earth^®^, Scotts-Sierra Horticultural Products Co., Maryville, OH, USA). Pots were maintained in a growth chamber at approx. 25°C with a photoperiod of 16 h light/d provided by fluorescent lamps with light intensities of between 315 and 325 μmol m^-2 ^s^-1^. Plants were fertilized with complete fertilizer solution (20:20:20 NPK + micronutrients) at two weeks after planting and every two weeks thereafter at a rate of 3 g l^-1 ^and 22 ml per pot.

Cell wall was extracted from 3-week old Eston leaflets following the protocol described by Eshel et al. [52]. One hundred gram of leaflets were ground in 100 mL of acid-methanol (4% v/v HCl) and filtered through Whatman filter paper number 1 (Whatman Ltd., Maidstone, UK). Residues were collected from the filter paper and blended twice more in 100 mL of methanol. This process was repeated until no phenols could be detected in the filtrate. The residues were blended three times in 100 mL of ice chilled acetone, and air dried overnight. The residues collected were then stored at -20°C until use as sole carbon source. Two grams mycelia were incubated on a rotary shaker at 125 rpm and 240 C in 2.5 L flasks containing 200 mL minimal salt broth (20 g/L D-glucose, 1 g/L KH_2_PO_4_, 0.5 g/L MgSO_4 _×7H_2_O, 0.5 g/L KCl, 0.15 g/L CaCl_2_×2H_2_O, 3 mg/L FeSO_4_× 7H_2_O, 3 mg/L ZnSO_4_×7H_2_O, 1.25 mg/L CuSO_4_×5H_2_O, 350 μg/L MnSO_4_×H_2_O and 250 μg/L Na_2_MoO_4_×2H_2_O) supplemented with 0.5% cell wall extract. After 72 hrs mycelia were collected and frozen in liquid nitrogen, and stored at -80°C until required.

### Fungal material

The *C. truncatum *isolate CT-21 used in this study was routinely maintained in Petri dishes containing oatmeal agar (30 g oatmeal flour, Quick Oats Robin Hood, Smucker Food of Canada, Markham, ON, Canada and 8.8 g granulated agar, Difco™, Becton, Dickinson & Company, Sparks, MD, USA] to 1 L of distilled water) at 22°C. For infection assays, conidia were harvested from 10- to 14-day old cultures, washed twice by centrifugation (3000 rpm, 5 minutes), and resuspended in sterile distilled water. For isolating total conidial RNA, conidia were pelleted and kept frozen until required.

For isolating total mycelial RNA, one mL of conidial suspension (4 × 10^-4 ^conidia mL^-1^) was incubated in complete medium (0.6% yeast extract, 0.3% acid casein hydrolysate, 0.3% enzymatic casein hydrolysate, 1% sucrose) at 22-25°C for 48 h with constant shaking at 150 rpm. Mycelia were harvested by filtering through nylon mesh and rinsed with distilled water twice. Collected mycelia were frozen in liquid nitrogen and stored at -80°C until required. For cell wall treated mycelia, collected mycelia were grown *in vitro *in minimal salt broth supplemented with Eston cell wall as sole carbon source at 22-25°C for 8 h with constant shaking at 150 rpm. Mycelia were harvested and stored as described above.

### RNA isolation and cDNA library construction

For library construction, leaflets of 3-week old lentil cultivar 'Eston' were harvested and inoculated with 15 μL droplets of conidial suspension (4 × 10^4 ^conidia mL^-1^) of *C. truncatum isolate *CT-21. Three hundred 6 mm diameter infection sites were punched from the leaflets using a cork borer during 48-72 hai and microscopically assessed under a compound microscope to ensure the correct timing for the biotrophy-necrotrophy switch. Leaflet discs (~200) with biotrophic primary hyphae and first indications of secondary hyphal growth signaling the necrotrophic phase, were frozen in liquid nitrogen and stored at -80°C until required.

Total RNA was isolated using phenol/chloroform extraction and LiCl precipitation [53]. Poly(A)^+ ^RNA (mRNA) was purified from total RNA using PolyATtract^® ^mRNA isolation system IV (Promega, Madison, WI, USA). A directional cDNA plasmid library was generated from 5 μg poly(A)^+ ^RNA using the pBluescript^® ^II XR cDNA library construction kit (Stratagene, La Jolla, CA, USA). The resulting cDNA library was ligated into the pBluscript II SK (+) vector using *Eco*RI and *Xho*I restriction sites transformed into XL10-Gold ultracompetent cells (Stratagene, La Jolla, CA, USA). The 5'-end single pass sequencing of cDNA inserts using M13 reverse universal primer was carried out in the DNA Technology Unit, Plant Biotechnology Institute, Saskatoon, Canada.

### EST analysis

ESTs were trimmed for vector, adaptor, and low quality sequence regions using DNAMAN software. The resulting EST sequences were queried against the NCBI non-redundant protein database using BLASTX algorithm http://www.ncbi.nlm.nih.gov/BLAST and against the Consortium for the Functional Genomics of Microbial Eukaryotes (COGEME) EST database http://cogeme.ex.ac.uk using TBLASTX algorithm [54].

An ORF finder algorithm http://www.ncbi.nlm.nih.gov/gorf was employed to predict coding region in all six frames *ab initio*; any sequence with a stop codon preceded by in-frame ATG codons was translated into protein sequence. All in-frame ATGs were considered as potential translation initiation sites while taking account of possible upstream ATG codons. The amino acid sequences were then scanned for potential SPs and transmembrane domain using SignalP server version 3.0 with default settings and iPSORT, and TMHMM server version 2, respectively. Proteins with predicted SPs were queried against the NCBI non-redundant protein database using BLASP algorithm with BlOSUM80 matrix without low complexity filter. Potential GPI-anchored proteins were identified using the BIG-PI Fungal Predictor http://mendel.imp.ac.at/gpi. Disulfide bonds in candidate effector proteins were predicted by SULFIND server http://disulfind.dsi.unifi.it. *N*-linked or *O*-linked glycosylation sites were predicted using NetNGlyc 1.0 and NetOGlyc 2.0 servers http://www.expasy.org.

### Infection time course assays

A detached leaf assay was used for semiquantitative RT-PCR. Leaflets from 3-week old Eston plants were detached and inoculated in Petri dishes lined with wet filter paper by droplet inoculation with *C. truncatum *isolate CT-21 as described before. Inoculated leaflets were incubated with 12 h photoperiod. The progress of *C. truncatum *infection was microscopically assessed and infection sites of leaflets were harvested as described above at 3 time points: appressorial penetration phase (16 hai), biotrophic stage (44 hai), and necrotrophic stage (68 hai). These leaflet discs were then frozen in liquid nitrogen until required.

### Microscopic evaluation of infection

Leaflet discs from all time points were fixed in a fixation solution (60% methanol, 30% chloroform, 10% acetic acid) until required. Fixed samples were rehydrated with decreasing ethanol gradients (100%, 80%, 70%, and 50% ethanol). Samples were then stained with 0.05% trypan blue (Harleco Parastains, Philadelphia, PA, USA) in distilled water overnight and destained in distilled water. The stained leaves were then mounted in 30% glycerol on glass slides. The developmental stages were examined and photographed using an epifluorescence microscope (Zeiss Axioplan, Jena, Germany). To investigate whether *C. truncatum *primary hyphae invaginate the host plasma membrane during the biotrophic phase of infection, leaflet discs from the biotrophic phase of infection were plasmolyzed and then stained with vital stain neutral red, and photographed using an epifluorescence microscope.

### Semiquantitative RT-PCR

Total RNA from each time point sample (conidia, mycelia, and cell wall treated mycelia [*in vitro *grown], and appressorial penetration, primary hyphae and secondary hyphae [*in planta*]) was isolated as described above. The genomic DNA was eliminated using RNase-free amplification grade DNase I (Invitrogen, Carlsbad, USA) from isolated total RNA and stored frozen at -80°C until required. Two micrograms of total RNA was reverse transcribed in a 20 μL reaction volume using 200 U SuperScript reverse transcriptase (Invitrogen, Carlsbad, CA, USA) following the protocol of the supplier. The resulting cDNA was diluted 20 times in sterilized ultrapure water. PCR amplification was performed with one microliter of cDNA from each sample as template using 28 cycles in an Eppendorf Mastercycler (Eppendorf, Hamburg, Germany). The PCR products were separated on 1% agarose gel and stained with ethidium bromide. The gene encoding 60S subunit of ribosmal protein (identified in the library) was used as control. All gene-specific primers used for RT-PCR analysis are listed in the additional file 4.

### Binary constructs and agroinfiltration

All primers and plasmids used in this study are described in additional file 5. For *in planta *expression of selected candidate effectors, including *M. oryzae *MgSM1, binary PVX constructs were made in vector pGR106 [55]. ORFs with and without SPs were cloned into pCR2.1 (Invitrogen) and sequenced. Confirmed fragments were digested with *Cla*I and *Not*I and ligated into pGR106. The constructs were then transformed to *A. tumefaciens *GV3101 carrying pSoup helper plasmid.

Infiltration assays with 0.3 OD_600 _of recombinant *A. tumefaciens *strains were performed on 4-6 weeks-old *Nicotiana tobacum *plants as described previously [38]. Responses were monitored from 2-10 days after infiltration. For autofluorescence detection, edges of lesions/infiltration zone were observed under UV light (480/40 nm excitation filter; 510 nm barrier) and photographed.

## Authors' contributions

VB constructed cDNA library and conducted EST analysis, expression profiling and functional analysis of candidate effectors, and drafted the manuscript. SB, AV and YW conceived the study, participated in its design and coordination, and helped to draft the manuscript. GS coordinated the sequencing aspects of the project. All authors read and approved the final manuscript.

## Supplementary Material

Additional file 1 **Fungal developmental stages**. Per cent of *in planta *fungal developmental stages in excised infected leaflet discs used for cDNA library construction. Click here for file

Additional file 2 **Clone IDs under various contigs**. NCBI GeneBank EST database ID to individual ESTs in each contig. Click here for file

Additional file 3 **Sequence alignment of CtCP1 with its orthologs**. ClustalW program was used to align *C. truncatum *CtCP1 with CP proteins present in other fungal species. Click here for file

Additional file 4 **List of primers (F, Forward; R, Reverse)**. Gene-specific primer sets were used to catalogue the expression profiles of CEAPs and candidate effectors. Click here for file

Additional file 5 **List of primers (F, Forward; R, Reverse)**. Primers were used to clone candidate effectors in the binary PVX-based expression vector pGR106. Click here for file
