# Supplementary material for: EST mining identifies proteins putatively secreted by the anthracnose pathogen Colletotrichum truncatum
Source: BMC Genomics. 2011 Jun 23;12:327. doi: 10.1186/1471-2164-12-327 (PMC3149586; doi:10.1186/1471-2164-12-327)
Supplement: Additional file 1 — Fungal developmental stages. Per cent of in planta fungal developmental stages in excised infected leaflet discs used for cDNA library construction. [file 1471-2164-12-327-S1.DOC]

| Stages | % |
| --- | --- |
| Appressorium penetration | 5 |
| Biotrophic stage | 20 |
| Early necrotrophic stage  (Switch stage) | 70 |
| Late necrotrophic stage | 5 |
