# Supplementary material for: EST mining identifies proteins putatively secreted by the anthracnose pathogen Colletotrichum truncatum
Source: BMC Genomics. 2011 Jun 23;12:327. doi: 10.1186/1471-2164-12-327 (PMC3149586; doi:10.1186/1471-2164-12-327)
Supplement: Additional file 2 — Clone IDs under various contigs. NCBI GeneBank EST database ID to individual ESTs in each contig. [file 1471-2164-12-327-S2.DOC]

| **Contigs** | **Clones** |
| --- | --- |
| Contig 1 | Ct21-90, Ct21-1988 |
| Contig 2 | Ct21-288, Ct21-156 |
| Contig 3 | Ct21-1168, Ct21-2343 |
| Contig 4 | Ct21-4886, Ct21-2273, Ct21-4841, Ct21-2075 |
| Contig 5 | Ct21-2920, Ct21-1881 |
| Contig 6 | Ct21-1002, Ct21-3652, Ct21-234, Ct21-527 |
| Contig 7 | Ct21-3813, Ct21-715, Ct21-943 |
| Contig 8 | Ct21-824, Ct21-3418 |
| Contig 9 | Ct21-4919, Ct21-2199 |
| Contig 10 | Ct21-4037, Ct21-3892 |
| Contig 11 | Ct21-1594, Ct21-2047 |
| Contig 12 | Ct21-2250, Ct21-2408 |
| Contig 13 | Ct21-128, Ct21-4906 |
| Contig 14 | Ct21-23, Ct21-2123, Ct21-3274 |
| Contig 15 | Ct21-1285, Ct21-2865, Ct21-4308 |
| Contig 16 | Ct21-59, Ct21-3965 |
| Contig 17 | Ct21-735, Ct21-4852, Ct21-4782 |
| Contig 18 | Ct21-4829, Ct21-975 |
| Contig 19 | Ct21-990, Ct21-1181 |
| Contig 20 | Ct21-2061, Ct21-1877 |
| Contig 21 | Ct21-2750, Ct21-2749 |
| Contig 22 | Ct21-3698, Ct21-3167 |
| Contig 23 | Ct21-1413, Ct21-4211 |
| Contig 24 | Ct21-4292, Ct21-4304 |
| Contig 25 | Ct21-4375, Ct21-4560 |
| Contig 26 | Ct21-4457, Ct21-74 |
| Contig 27 | Ct21-3775, Ct21-4670 |
| Contig 28 | Ct21-924, Ct21-1307 |
| Contig 29 | Ct21-4776, Ct21-1400 |
| Contig 30 | Ct21-2184, Ct21-3340 |
| Contig 31 | Ct21-406, Ct21-3561 |
| Contig 32 | Ct21-1705, Ct21-1961 |
