# Supplementary material for: EST mining identifies proteins putatively secreted by the anthracnose pathogen Colletotrichum truncatum
Source: BMC Genomics. 2011 Jun 23;12:327. doi: 10.1186/1471-2164-12-327 (PMC3149586; doi:10.1186/1471-2164-12-327)
Supplement: Additional file 3 — Sequence alignment of CtCP1 with its orthologs. ClustalW program was used to align C. truncatum CtCP1 with CP proteins present in other fungal species. [file 1471-2164-12-327-S3.DOC]

CtCP1 MQLSGLVTFLSSVAAAAAVTVSYDTGYDDGSRSLTSIACSDGANGLISRYGWQNQGQVAR 60

MgSM1 MQFSNILSIFTLAAAAQAVSVSYDTGYDDGSRSLTAVSCSDGANGLITKYGWQTQGQIRN 60

DQ464903 MQFSNLFKLALFTAAVSADTVSYDTGYDDASRSLTVVSCSDGANGLITRYHWQTQGQIPR 60

DQ494198 MQLSNIFTLALFTAAVSADTVSYDTGYDNGSRSLNDVSCSDGPNGLETRYHWSTQGQIPR 60

AY826795 MKFTGILSALALTSAVSATTVSYDTGYDDKSRPMTSVACSDGSNGLITKYGWKTQGNIP- 59

*:::.:.. .:*. * :********: **.:. ::****.*** ::* *..**::

CtCP1 FPYIGGADAIAGWNSPNCGTCWQLTYRGKSINVLAVDHAGSGFNIALGALNDLTNGQAAQ 120

MgSM1 FPYIGGVDAVGGWNSPSCGTCWQLTYNGKSINVLAIDHA-SGFNIGLAAMNDLTNGQAGS 119

DQ464903 FPYIGGVQAVAGWNSPSCGTCWKLTYSGKTIYVLAVDHSAAGFNIGLDAMNALTNGNAVQ 120

DQ494198 FPYIGGVAAVAGWNSANCGTCWKLQYSGHTIYVLAVDHAASGFNIALDAMNALTGGQAVK 120

AY826795 TPYVGGVNIIAGWNSPNCGGCYRLEFKGRKINVLAIDHAASGFNIGLDAMNALTGGQATQ 119

**:**. :.****..** *::* : *:.* ***:**: :****.* *:* **.*:* .

CtCP1 LGRIDAQATQVGLNACGL-- 138

MgSM1 LGRIEAQSQQVGLNACGL-- 137

DQ464903 YGRVDATASQVAVSNCGL-- 138

DQ494198 LGRVSATATQVPVKNCGL-- 138

AY826795 LGRINAQVYHADASACGLKK 139

**:.* :. . ***

Putative signal peptide at the N-terminus is lined above the sequence.

Four conserved cysteine residues are indicated by filled triangles.
