# Supplementary material for: EST mining identifies proteins putatively secreted by the anthracnose pathogen Colletotrichum truncatum
Source: BMC Genomics. 2011 Jun 23;12:327. doi: 10.1186/1471-2164-12-327 (PMC3149586; doi:10.1186/1471-2164-12-327)
Supplement: Additional file 4 — List of primers (F, Forward; R, Reverse). Gene-specific primer sets were used to catalogue the expression profiles of CEAPs and candidate effectors. [file 1471-2164-12-327-S4.DOC]

| **Primer name** | **Oligo sequence (5’ 3’)** |
| --- | --- |
| *60S (C. truncatum)*  *60S (L. culinaris)* | TCGTTGTTGGTCTTGTGGG (F) ATCTTGTGGTTGACAGAGGT (R)  GCGGCGTTTCTGATTCAA (F) GCATTTACACCATCGGACTC (R) |
| Candidate effector genes | |
| *CtCP1* | CCGTCACCTTCCTCTCTTC (F)  TTGGTGAGGTCGTTCAG (R) |
| *Contig 32* | TTCACCGCCGTCCTCTCTT (F)  ACAGCGTTGGGCTCGTT (R) |
| *Ct21-4630* | TGTCCGCCACTACCCTTCT (F) AACGAGTCCCAGGTGAGGA (R) |
| *Contig 5* | ATGCGTTCTTCCTCCCT (F)  GTCTGGGTTCTTGATGTTG (R) |
| *Ct21-1573* | ACCATCATCTCGCAGAA (F)  ACAGACGGGAATCTTGA (R) |
| *Contig 6* | GGCTCCGTCCTTGAGAA (F)  GTCCTCGCAGTAGTTGA (R) |
| *Ct21-741* | ACGGGAAGGGATTTGGG (F)  AAGAGGTCGTGGTTGGC (R) |
| *Ct21-1631* | CCCTTGCGTCCAGATTGA (F)  ACCATTACCAGATGCGGC (R) |
| *Contig 10* | CGACAGCGTCAGCACCTCACA (F)  GGAGACCGAGCAGAAGTGGGAA (R) |
| CIH1 and GPI-anchored protein encoding genes | |
| *Ct21-2424* | TCCATCTTCACCGTCCT (F) TGGTCGTCAAGGTCTGG (R) |
| *Ct21-2435* | TCCACAAGACCACCGTCAAG (F) AAGGGCTTCAGGCAAGAGGT (R) |
| *Ct21-3485* | CTTTGCTGTTGCTCTCC (F) AGTCAGTGGTGATACCG (R) |
| *Ct21-90* | ACACTCTGGTTCGTCAAGC (F) GGCAGTTGTTGTAGCAGGT (R) |
| *Ct21-156* | CCCATCTTCAAGCCCAC (F) TTGGCAGACAGGGTAAC (R) |
| *Ct21-2343* | GCCTCACCTACCCTGAA (F) CTGCTCCTTGACCGTGT (R) |
| *Ct21-2075* | CGGTGTCCCTATCAAGTC (F) TGACGGTCTCGTAGATGA (R) |
| *Ct21-949* | GGTATCACCCTTCCCAACCT (F) TGAAGACATCGCAAGAGGTG (R) |
| *Ct21-1020* | AGAGAGCGAAAGCAGCAGAC (F) GCAGAAGAGACCCGAGAGTG (R) |
| *Ct21-3268* | GTACTCTTACGTCCTCCT (F) AGAGCGATCATACCGAA (R) |
