# Supplementary material for: EST mining identifies proteins putatively secreted by the anthracnose pathogen Colletotrichum truncatum
Source: BMC Genomics. 2011 Jun 23;12:327. doi: 10.1186/1471-2164-12-327 (PMC3149586; doi:10.1186/1471-2164-12-327)
Supplement: Additional file 5 — List of primers (F, Forward; R, Reverse). Primers were used to clone candidate effectors in the binary PVX-based expression vector pGR106. [file 1471-2164-12-327-S5.DOC]

| **Name** | **Sequence (5’→3’)** | **Plasmid** |
| --- | --- | --- |
| pGR106Contig6F | CA*ATCGAT*ATGAAGGCCGCCGTCATCCT | pGR106-Contig6 |
| pGR106Contig6R | GT*GCGGCCGC*TTACAAGTCCTCGCAGTAGT |
| pGR106Contg6-SPF | CA*ATCGAT*TCCGTCCTTGAGAAGCGCGA | pGR106-Contig6-SP* |
| pGR106CtCP1F | CA*ATCGAT*ATGCAGCTCTCCGGCCTCGT | pGR106-Contig8 |
| pGR106CtCP1R | GT*GCGGCCGC*TACAGGCCACAGGCGTTAA |
| pGR106CtCP1-SPF | CA*ATCGAT*TCCGACGGCGCCAACGGCCTC | pGR106-Contig8-SP* |
| pGR106Contig32F | CA*ATCGAT*ATGAAGTTCACCGCCGTCCT | pGR106-Contig32 |
| pGR106Contig32R | GT*GCGGCCGC*TTACTCGAGGTTGAAAGCGA |
| pGR106Contg32-SPF | CA*ATCGAT*GCCATCAGCAAG CGTGACGT | pGR106-Contig32-SP* |
| pGR106-MgSM1F | CA*ATCGAT*ATGCAGTTCTCCAACATCCTC | pGR106-MgSM1 |
| pGR106-MgSM1R | GT*GCGGCCGC*TTACAGGCCGCAGGCGTTGAG |
| pGR106MgSM1-SPF | CA*ATCGAT*GTCAGCGTCTCATACGACACC | pGR106-MgSM1-SP* |
| pGR106MgSM1-SPF | CA*ATCGAT*TCATACGACACCGGCTACGAC | pGR106MgSM1-SP* |

Restriction sites are italicized. Forward primers contain *Cla*I whereas *Not*I restriction sites are present in reverse primers.

*Reverse primers (R) are the same as the primers used to clone ORF of corresponding effectors with SPs.
